# Risk factors for fluorouracil-induced cardiotoxicity in patients with gastrointestinal tumor

**DOI:** 10.3389/fcvm.2025.1515509

**Published:** 2025-02-05

**Authors:** Yuting Wu, Chen Lv, Jindong Li, Ying Ma, Xiaoli Zhu

**Affiliations:** ^1^ Department of Pharmacy, The Affiliated Taizhou People’s Hospital of Nanjing Medical University, Taizhou, Jiangsu, China; ^2^ Department of Medical Oncology, The People’s Hospital of Hengshui, Hengshui, Hebei, China; ^3^ Department of Pharmacy, The People’s Hospital of Hengshui, Hengshui, Hebei, China

**Keywords:** fluorouracil drugs, chemotherapy, gastrointestinal neoplasms, cardiotoxicity, risk factors

## Abstract

**Objective:**

To explore the risk factors for cardiotoxicity in patients with gastrointestinal (GI) tumors treated with fluorouracil drugs.

**Methods:**

This study included patients with GI tumors who received fluorouracil at our hospital between January 2018 and April 2022. The demographic and clinical characteristics were collected. The risk factors associated with the cardiotoxicity of fluorouracil were explored using multivariable logistic regression.

**Results:**

A total of 300 patients were finally included and divided into the cardiotoxicity (*n* = 81) and non-cardiotoxicity groups (*n* = 219). The occurrence of fluorouracil-induced cardiotoxicity was higher in patients with hypertension, hyperlipidemia, diabetes mellitus, older age, those treated with capecitabine, and combined radiotherapy. The multivariable logistic regression showed that treatment with capecitabine, history of hyperlipidemia, history of diabetes, older age, and combined radiotherapy were independent risk factors for the cardiotoxicity of fluorouracil.

**Conclusion:**

Hyperlipidemia, diabetes, older age, treatment with capecitabine, and adjuvant radiotherapy might be independent risk factors for the cardiotoxicity of fluorouracil in patients with GI tumors.

## Introduction

Gastrointestinal (GI) cancer is a term for a group of cancers that includes tumors of the colon, rectum, stomach, pancreas, esophagus, anus, gallbladder, liver, and bile duct. GI cancers account for 26% of global cancers and are responsible for 35% of all cancer-related deaths (1). Colorectal cancer (CRC) is the most common GI tumor and the third leading cause of cancer-related death globally (2). Surgery, chemotherapy, radiation therapy, targeted therapy, and immunotherapy are standard treatment approaches for these malignancies (3). Chemotherapy can be given before or after surgery or as the main treatment; neoadjuvant treatment usually reduces the size of the tumor, and as such, it may facilitate surgery (4). It also might exterminate cancer areas that cannot be completely removed with surgery, which helps prevent cancer recurrence and prolong survival time (5). Fluorouracil drugs (5-fluorouracil, capecitabine, and S-1) are the cornerstone of chemotherapy regimens for GI tumors. S-1 refers to a novel oral fluorouracil antitumor drug that combines three pharmacological agents: tegafur (FT), a prodrug of 5-fluorouracil (5-FU), 5-chloro-2,4-dihydroxypyridine (CDHP), an inhibitor of dihydropyrimidine dehydrogenase, and potassium oxonate (Oxo), a reducer of GI toxicity (6). They are the second most commonly used cardiotoxic drugs after anthracyclines (7), acting as an antimetabolite agent with an important role in cancer treatment. Their structure is similar to that of enzymes for deoxyribonucleic acid (DNA) replication, and they are activated to inhibit the synthesis of DNA and ribonucleic acid (RNA) after cellular uptake (8). Yet, their effectiveness is limited by drug resistance (9). Also, they can lead to cardiotoxicity. The main manifestations of fluoropyrimidine-related cardiotoxicity are chest tightness, palpitation (10), and angina pectoris (11). Less common cardiotoxic manifestations include atrial fibrillation (12, 13), arrhythmias (14, 15), myocarditis (14, 16), pericarditis (17), heart failure (14), and even death (18, 19). Also, fluorouracil-related cardiotoxicity can sometimes be fatal and affect the disease prognosis, and certain risk factors, such as comorbidities, might increase the incidence of cardiotoxicity (20). Chao et al. (21) performed a systematic review and meta-analysis to assess risk factors of fluoropyrimidine-induced cardiotoxicity among cancer patients. They discovered that those with cardiac diseases, hypertension, and smoking have higher risks. They concluded that risk assessment is essential to help mitigate risk and improve patient outcomes. Yet, the inconsistency in risk factor identification for fluorouracil-induced cardiotoxicity in patients with GI tumors prevents the development of predictive modeling of the risk of cardiotoxicity for GI patients.

This study further explored the risk factors for fluorouracil-induced cardiotoxicity in patients with GI tumors.

## Materials and methods

### Study design and patients

This retrospective case-control study included patients with GI cancer who received fluorouracil chemotherapy at our hospital between January 2018 and April 2022. The inclusion criteria were (1) age >18 years old, (2) GI tumor (esophageal, gastric, pancreatic, liver, bile duct, duodenum, gallbladder, and CRC) confirmed by histopathology, and (3) treated with fluorouracil drugs. The exclusion criteria were (1) participation in other clinical trials, (2) no cardiac monitoring, (3) heart disease (such as coronary heart disease, valvular heart disease, and cardiomyopathy) confirmed before diagnosis or chemotherapy, (4) history of other malignant tumors, (5) neoadjuvant chemotherapy or palliative chemotherapy for metastatic cancer, or (6) incomplete data.

**Figure 1:**
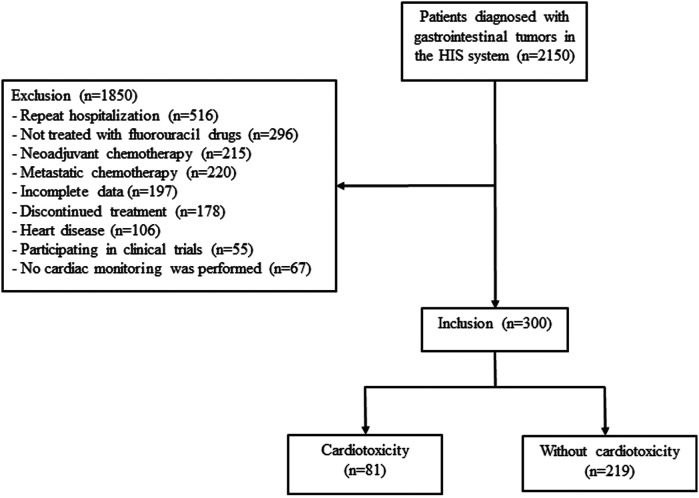
Study flowchart for patients selection and exclusion.

The patients were divided into the cardiotoxicity and non-cardotoxicity groups according to whether cardiotoxicity of fluorouracil occurred (see Figure 1).

This study was approved by the ethics committee of Hengshui People's Hospital (No. KY 2020-188-01). This study was a retrospective study. Therefore, the ethics committee of Hengshui People's Hospital waived the requirement to obtain distinct written informed consent from the patients.

### Data collection

In our study, a meticulous collection of clinical data was performed, encompassing a spectrum of parameters: gender, age, body mass index (BMI), body surface area, tumor type, and tumor stage. The history of comorbidities, including hypertension, hyperlipidemia, and diabetes, along with lifestyle factors such as smoking and alcohol consumption, were also collected. Additionally, laboratory examinations before the first cycle of fluorouracil chemotherapy, covering renal function, cardiac function, hematological indicators, liver function, and electrolyte levels, were also collected. Renal function was meticulously assessed through the estimated glomerular filtration rate (eGFR), serum creatinine, and blood urea nitrogen. Cardiac function was evaluated using N-terminal pro-B-type natriuretic peptide (NT-proBNP)/B-type natriuretic peptide (BNP), troponin, creatine kinase-MB (CK-MB), and left ventricular ejection fraction (LVEF) values. Hematological indicators included hemoglobin, platelets, and white blood cells, while liver function was gauged by transaminases, bilirubin, and albumin levels. Electrolyte levels, including potassium, sodium, calcium, and magnesium, were also monitored. Additionally, data on adjuvant treatments with radiotherapy, fluorouracil, and other chemotherapy drugs were meticulously documented.

The clinical pharmacist and chief physician evaluated the cardiotoxicity of fluorouracil according to the medical records. Cardiotoxicity of fluorouracil was considered when at least one of the following criteria was met (20, 22): (1) abnormal in electrocardiogram (ECG); (2) abnormal elevation of myocardial enzymes and markers; (3) LVEF decreased by at least 10% to absolute value <55%. The chief physician decided that heart diseases caused by other drugs were not to be considered cardiotoxicity of fluorouracil. Cardiotoxicity was observed during the fluorouracil treatment cycle and 6 months after chemotherapy.

Two experienced ECG physicians analyzed the collected ECGs to identify abnormalities based on the 9th edition of Internal Medicine. The following abnormalities were included: sinus tachycardia, sinus bradycardia, ventricular premature beats, junctional premature beats, atrioventricular block, right bundle branch block, ST-segment changes (elevation or depression), and T-wave abnormalities (T-wave inversion or flattened T-wave). The detailed criteria and the normal ranges for cardiac enzymes are provided in the Supplementary Material.

When assessing cardiotoxicity based on patient information, any discrepancies were solved by a multidisciplinary team (MDT), which included the following specialists at the level of associate chief physician or above: medical oncologists, cardiologists, ECG specialists, ultrasound specialists, laboratory medicine specialists, and clinical pharmacists specializing in oncology.

### Sample size

The sample size was determined based on the 10 events per variable (EPV) principle, which is a heuristic often used in logistic regression analysis to ensure adequate statistical power and to prevent overfitting. This principle suggests that at least 10 events for each independent variable should be included in multivariable logistic regression analysis.

### Statistical analysis

All statistical analyses were performed using SPSS 25.0 (IBM Corp., Armonk, NY). The continuous variables conforming to normal distribution were described as mean with standard deviation (SD) and compared using the independent-sample t-test. The continuous variables conforming to a skewed distribution were described as median (Q1, Q3) and compared using the Wilcoxon signed-rank test. Categorical data were described as *n* (%) and compared using the chi-squared test. The risk factors for cardiotoxicity of fluorouracil were determined by binary multivariable logistic regression analysis, and variables with *P* < 0.05 in the univariable analysis were included in the multivariable analysis. The Pearson correlation analysis and variance inflation factor (VIF) were used to assess the correlation and collinearity of characteristics included in the correlation analysis and collinearity. The age was converted into a binary variable based on the median (63 years) to improve the interpretability of the model. Considering the potential impact of the cutoff on the results, a sensitivity analysis was also conducted using a cutoff of 60 years. A two-sided *P* < 0.05 represented statistical significance.

## Results

### Patient characteristics

Among 2,150 patients diagnosed with GI tumors between January 2018 and April 2022, 1,850 were excluded for repeated hospitalization (*n* = 516), not treated with fluorouracil drugs (*n* = 296), discontinued treatment (*n* = 178), underwent neoadjuvant chemotherapy (*n* = 215) or metastatic chemotherapy (*n* = 220), heart disease (*n* = 106), participated in clinical trials (*n* = 55), or no cardiac monitoring (*n* = 67) (Figure 1). A total of 300 patients (181 female) were finally included, and cardiotoxicity occurred in 81/300 (27.00%) patients after a median of 6.37 (2–13) cycles. Discrepancies in the diagnosis of cardiotoxicity were observed between the two physicians in 11 patients.

A total of 81 patients with cardiotoxicity showed abnormal ECG changes, including 46 ST segment changes (56.79%), 24 T wave changes (29.63%), 16 sinus bradycardia (19.75%), 7 sinus tachycardia (8.64%), 6 ventricular or borderline premature beats (7.41%), and 8 (9.88%) atrioventricular block or right bundle branch block. Fifteen patients in the cardiotoxicity group showed increased myocardial enzyme profile and myocardial markers, and 2 patients showed decreased LVEF. Due to the occurrence of cardiotoxicity, the dose of fluorouracil was reduced by 80% in 2 cases. In contrast, the chemotherapy regimen and drug dose were unchanged in the remaining 79 patients, and no special drug therapy was given for cardiotoxicity.

Compared to patients without cardiotoxicity, patients with cardiotoxicity were older (64.59 ± 8.56 vs. 60.49 ± 10.67 years old, *P* = 0.001), had higher rates of hypertension (38.27% vs. 19.63%, *P* = 0.001), hyperlipidemia (25.93% vs. 5.48%, *P* < 0.001), and diabetes (25.93% vs. 7.31%, *P* < 0.001), and received combined radiotherapy more often (58.02% vs. 15.98%, *P* < 0.001). In addition, significant differences were observed in fluorouracil drugs between the two groups (*P* = 0.001) (Table 1). The laboratory examinations conducted before the first cycle of fluorouracil chemotherapy, including renal function, cardiac function, blood routine, liver function, and electrolyte levels, showed no statistically significant differences (Supplementary Table S1).

**Table 1:** Comparison of basic data of patients with and without cardiotoxicity.

Characteristics	Non-cardiotoxicity group (*n* = 219)	Cardiotoxicity group (*n* = 81)	*P*
Age (years)			0.004
≤63	119 (54.34)	29 (35.80)	
>63	100 (45.66)	52 (64.20)	
Gender (female)	131 (59.82)	50 (61.73)	0.764
Hypertension	43 (19.63)	31 (38.27)	0.001
Hyperlipidemia	12 (5.48)	21 (25.93)	<0.001
Diabetes	16 (7.31)	21 (25.93)	<0.001
Smoking	13 (5.94)	4 (4.94)	0.740
Drinking	12 (5.48)	4 (4.94)	0.853
Tumor types			0.555
Upper gastrointestinal	103 (47.03)	35 (15.98)	
Lower gastrointestinal	116 (52.97)	46 (56.79)	
Tumor staging			0.139
Ⅱ	57 (26.03)	19 (23.46)	
Ⅲ	111 (50.68)	34 (41.98)	
Ⅳ	51 (23.29)	28 (34.57)	
Fluorouracil drugs			0.001
S1	38 (17.35)	8 (9.88)	
5-fluorouracil	121 (55.25)	32 (39.51)	
Capecitabine	60 (27.40)	41 (50.62)	
Combined oxaliplatin	175 (79.91)	58 (71.60)	0.125
Combined platinum	11 (5.02)	7 (8.64)	0.241
Combined taxanes	26 (11.87)	9 (11.11)	0.855
Combined antiangiogenic	47 (21.46)	24 (29.63)	0.139
Combined immunotherapy	17 (7.76)	9 (11.11)	0.360
Combined irinotecan	20 (9.13)	12 (14.81)	0.157
Combined radiotherapy	35 (15.98)	47 (58.02)	<0.001
BMI (kg/m^2^)			0.863
<18.5	34 (15.53)	13 (16.05)	
18.5–24.9	141 (64.38)	51 (62.96)	
25.0–29.9	41 (18.72)	17 (20.99)	
>29.9	3 (1.37)	0	
BSA	1.67 ± 0.17	1.66 ± 0.18	0.715
Dosage			
5-fluorouracil (mg/m^2^)			0.052
≥2,500	20 (9.13)	10 (12.35)	
2,000–2,499	21 (9.59)	9 (11.11)	
1,400–1,999	65 (29.68)	9 (11.11)	
Capecitabine (mg/m^2^/d)			0.296
≥1,800	25 (11.42)	13 (16.05)	
1,500–1,799	20 (9.13)	18 (22.22)	
1,000–1,499	13 (5.94)	6 (7.41)	
<1,000	2 (0.91)	4 (4.94)	
S1 (mg/m^2^/d)			0.890
≥890	22 (10.05)	4 (4.94)	
119–100	5 (2.28)	1 (1.23)	
<100	11 (5.02)	3 (3.70)	

BMI, body mass index; BSA, body surface area.

### Risk factors

**Figure 2:**
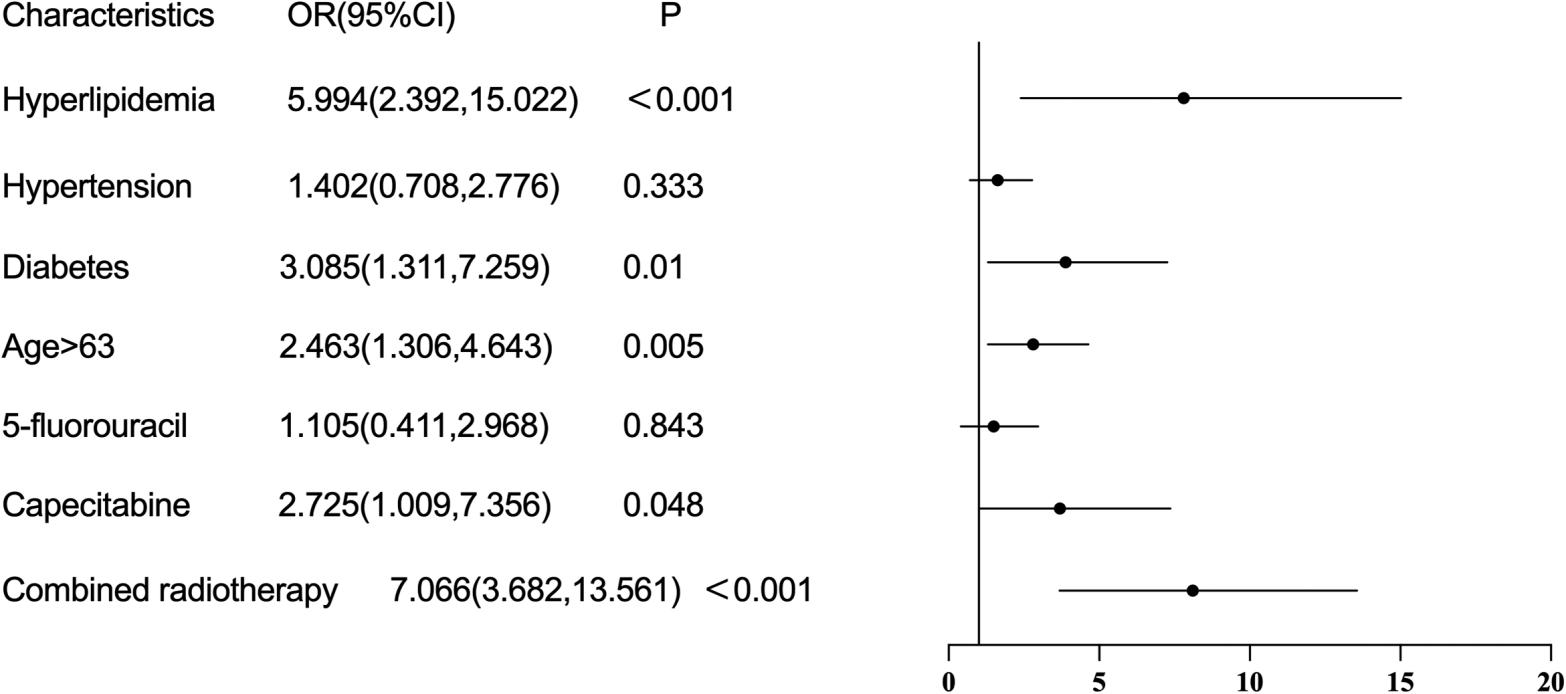
Risk factors of cardiotoxicity caused by fluorouracil drugs.

Multivariable logistic regression analysis showed that treatment with capecitabine (OR = 2.725, 95%CI: 1.009–7.356, *P* = 0.048), history of hyperlipidemia (OR = 5.994, 2.392–15.022, *P* < 0.001), history of diabetes (OR = 3.085, 95%CI: 1.311–7.259, *P* = 0.010), older age (OR = 2.463, 95%CI: 1.306–4.643, *P* = 0.005), and combined radiotherapy (OR = 7.066, 95%CI: 3.682–13.561, *P* < 0.001) were risk factors for the occurrence of cardiotoxicity of fluorouracil (Figure 2). The sensitivity analysis indicated similar results. Treatment with capecitabine, history of hyperlipidemia, history of diabetes, older age, and combined radiotherapy were identified as risk factors for the occurrence of cardiotoxicity related to fluorouracil (Table 2). The Pearson correlation analysis in Table 3 indicated that the characteristics included in the multivariable logistic regression analysis had either weak or no statistically significant correlations. Supplementary Table S2 displays the tolerance and VIF results for the collinearity analysis, indicating the multicollinearity was within acceptable limits. There were 6 independent variables included in the regression analysis, requiring a minimum of 60 events with cardiotoxicity. The sample size in our study met this requirement.

**Table 2:** Risk factors of cardiotoxicity caused by fluorouracil drugs (sensitivity analysis).

	95%CI	*P*
Hyperlipidemia	8.084 (3.064, 21.334)	<0.001
Hypertension	1.454 (0.718, 2.941)	0.298
Diabetes	3.851 (1.614, 9.191)	0.002
Age (>60 years)	1.045 (1.012, 1.079)	0.007
Fluorouracil drugs		
S1	REF	REF
5-fluorouracil	1.707 (0.552, 5.282)	0.353
Capecitabine	5.403 (1.689, 17.283)	0.004
Combined radiotherapy	9.582 (4.867, 18.866)	<0.001

**Table 3:** Pearson correlation analysis.

Variable	Hypertension	Hyperlipidemia	Diabetes	Age	Fluorouracil drugs	Combined radiotherapy
Hypertension	1	0.095	0.209^a^	0.163^a^	−0.178^a^	0.083
Hyperlipidemia	0.095	1	0.225^a^	0.006	−0.116^b^	0.047
Diabetes	0.209^a^	0.225^a^	1	0.066	−0.027	0.043
Age	0.163^a^	0.006	0.066	1	0.052	0.022
Fluorouracil drugs	−0.178^a^	−0.116^b^	−0.027	0.052	1	−0.094
Combined radiotherapy	0.083	0.047	0.043	0.022	−0.094	1

^a^ Significant correlation at the 0.01 level (two-tailed test).

^b^ Significant correlation at the 0.05 level (two-tailed test).

The subsequent analyses are shown in the Table 4. Cardiotoxicity was observed during the fluorouracil treatment cycle and 6 months after chemotherapy. Cardiotoxicity diagnosed based on abnormal ECG and elevation of myocardial enzymes and markers was the highest in patients treated with capecitabine (*n* = 41 and *n* = 9, respectively), followed by those treated with 5-fluorouracil (*n* = 32 and *n* = 5, respectively) and S1 drugs (*n* = 8 and *n* = 1, respectively). Also, cardiotoxicity was seen earlier in patients treated with capecitabine (4.59 ± 2.09) and S1 drugs (4.38 ± 2.33) than those treated with 5-fluorouracil (5.50 ± 2.77).

**Table 4:** Time and diagnosis reason for cardiotoxicity.

	5-fluorouracil	Capecitabine	S1	*P*
Time of cardiotoxicity (treatment cycle)	5.50 ± 2.77	4.59 ± 2.09	4.38 ± 2.33	0.221
Diagnosis of cardiotoxicity				0.001
Abnormal ECG changes	32	41	8	
Elevation of myocardial enzymes and markers	5	9	1	
LVEF	1	1	0	

ECG, electrocardiogram; LVEF, left ventricular ejection fraction

## Discussion

The present study revealed that hyperlipidemia, diabetes, older age, and combined radiotherapy were risk factors for the cardiotoxicity of fluorouracil in patients with GI tumors. These results provide relevant information for applying fluorouracil in patients with GI tumors and early prediction of cardiotoxicity.

The cardiotoxicity rate of 27% observed in the present study deviates from that reported in previous literature (23, 24). This discrepancy may arise from varying definitions of cardiotoxicity; for instance, Johannes et al. limited their definition to include only cardiac ischemia/infarction, heart failure, atrial fibrillation, and other arrhythmias (25). Our study encompassed a broader spectrum of patients, including those exhibiting sinus bradycardia and tachycardia, as we posit that any chemotherapy-related cardiac events should be classified as cardiotoxic. Notably, only 20% of patients in the cardiotoxicity group presented with a significant increase in myocardial enzyme profile and myocardial markers. The potential for cardiac enzymes or myocardial markers to be either elevated or within normal limits suggests that cardiotoxicity associated with 5-fluorouracil may not invariably result in myocardial necrosis.

Previous studies have reported that the incidence of cardiotoxicity of fluorouracil drugs is about 1%–39%, while that of fatal cardiotoxicity is about 0%–13% (26). The recognized mechanisms include coronary artery spasm, autoimmune-mediated myocardial injury, direct myocardial necrosis, vascular endothelial dysfunction, and thrombosis caused by hypercoagulability (6, 11, 27). According to the previous study (28), 16%–50% of patients with malignant tumors treated with 5-fluorouracil presented with abnormal ECG changes, mainly manifesting as ST-T changes and arrhythmias. However, their myocardial enzyme levels were usually normal, and most cardiotoxic events caused by 5-fluorouracil occurred in the first cycle of treatment or shortly after the beginning of the treatment (29). In our study, most of the patients were diagnosed according to abnormal ECG changes, which was consistent with previous studies.

Building on the findings of Peng et al. (30) and the distinct cardiotoxicity profiles of fluoropyrimidines, our study adds to the body of evidence suggesting differential cardiotoxic potential among 5-fluorouracil, capecitabine, and S1. The higher incidence of cardiotoxicity with capecitabine, as compared to 5-fluorouracil and S1, may reflect its unique metabolic activation and subsequent impact on cardiac function (31). However, it remains unclear why treatment with capecitabine is more likely to cause cardiotoxicity. Drawing from the mechanistic insights provided in recent literature, including the comprehensive review by Kanduri *et al*. on fluoropyrimidine-associated cardiotoxicity and the prospective study by Lestuzzi *et al*. on capecitabine-related cardiotoxicity during physical exercise (32, 33), our understanding of the cardiotoxic effects of capecitabine is further informed. The study by Lestuzzi *et al*. observed a higher incidence of cardiotoxicity with capecitabine, noting an increased risk of myocardial ischemia and arrhythmias, particularly in the context of physical exertion (33). It underscores the need for further exploration of capecitabine's metabolic pathways, its impact on cardiac function, and potential interactions with other risk factors. Cardiotoxicity should be especially considered in patients treated with capecitabine chemotherapy compared to the GI tumor patients treated with fluorouracil and S1. If serious cardiotoxic events occur, the drugs must be discontinued or changed (34).

In a retrospective study, 72% of patients who developed cardiotoxicity after 5-fluorouracil were >55 years old (35). Nonetheless, age did not result as an independent risk factor for the cardiotoxicity of fluorouracil in some previous studies (36). Our findings suggested that age ≥63 years old may be an independent risk factor for the cardiotoxicity of fluorouracil. Therefore, older age might be a risk factor.

Several studies have reported that underlying diseases, such as hypertension, diabetes, and hyperlipidemia, are significantly correlated with the incidence of cardiotoxicity of fluorouracil (32, 37). Our results also suggested that pre-existing hyperlipidemia and diabetes could significantly increase the cardiotoxicity of fluorouracil. In univariable analysis, hypertension was significantly associated with cardiotoxicity. This correlation was not observed in the logistic regression model, possibly due to the collinearity between hypertension and other risk factors.

Whether previous or concurrent radiation therapy is an independent risk factor for the cardiotoxicity of fluorouracil remains unclear. Some studies have reported a lack of association between the history of thoracic radiation therapy and an increased risk of cardiotoxicity of fluorouracil (38). However, a previous study (26) reported radiation therapy as a risk factor for patients treated with fluorouracil. Our results showed that concurrent radiotherapy increased the incidence of cardiotoxicity in patients with GI tumors receiving fluorouracil chemotherapy. Also, the incidence of cardiotoxicity in patients treated with combined radiotherapy was higher than in patients who did not receive combined radiotherapy. It has been reported that the cardiotoxicity of fluorouracil partially occurs due to small vessel thrombosis. Fluorouracil acts as a radiosensitizer when used with radiation therapy (35), which might also be related to cardiotoxicity. Therefore, it is recommended that heart-related examinations be diligently performed in patients undergoing combined radiotherapy and chemotherapy for gastrointestinal tumors. Upon detecting cardiotoxicity, consideration should be given to the potential need for drug withdrawal and the implementation of appropriate cardiac protection strategies (34).

Our findings underscore the importance of early identification of fluorouracil-induced cardiotoxicity in patients with gastrointestinal tumors. Implementing risk scores, as highlighted in the recent ESC guidelines, could significantly enhance the ability to predict and manage cardiotoxicity (39). These tools provide a structured approach to risk stratification, allowing clinicians to make informed decisions regarding treatment adjustments and cardiac monitoring. By incorporating such scores into clinical practice, we can potentially reduce the incidence of severe cardiotoxic events and improve patient outcomes. Future research should build on this approach, further exploring and validating the utility of risk assessment strategies in the context of fluorouracil chemotherapy.

Our study highlights the necessity for stringent cardiac surveillance in patients receiving fluorouracil drugs, particularly capecitabine, due to its association with increased cardiotoxicity risks. It is especially pertinent for patients with comorbidities like hypertension, hyperlipidemia, and diabetes, where proactive cardiac management could significantly enhance treatment outcomes. The higher incidence of cardiotoxicity with treatment with capecitabine, as indicated by Lestuzzi *et al*.'s study (33), which noted a rise in myocardial ischemia and arrhythmias during physical exertion, warrants further investigation into its metabolic effects on cardiac function and interactions with other risk factors. Integrating these insights with the existing body of knowledge on cardiotoxicity's pathophysiology, we reinforce the importance of developing personalized strategies to prevent and manage chemotherapy-induced cardiotoxicity in cancer patients.

The present study has several limitations. Firstly, as a retrospective study, the criteria for cardiotoxicity were based on an electrocardiogram, myocardial enzyme spectrum, myocardial markers, and ECG, which may have led to omissions of cardiotoxicity cases presenting only with clinical symptoms without abnormal evaluation indexes. Secondly, the study did not include tegafur, a drug rarely used in our hospital setting. Additionally, the non-significant results regarding the time of toxicity between capecitabine and 5-FU may be attributed to the sample size, which could have been too small to detect a significant difference in toxicity manifestation times. Moreover, patients undergoing neoadjuvant therapy and those with metastases were excluded due to the heterogeneity of their treatment protocols. Addressing these limitations in larger, prospective studies would be beneficial.

In conclusion, treatment with capecitabine, hyperlipidemia, diabetes, older age, and combined radiotherapy are risk factors for cardiotoxicity in GI patients treated with fluorouracil drugs.

## Data Availability

The original contributions presented in the study are included in the article/Supplementary Material, further inquiries can be directed to the corresponding author.
