# Supplementary material for: Risk factors for fluorouracil-induced cardiotoxicity in patients with gastrointestinal tumor
Source: Front Cardiovasc Med. 2025 Feb 5;12:1515509. doi: 10.3389/fcvm.2025.1515509 (PMC11835890; doi:10.3389/fcvm.2025.1515509)
Supplement: Supplementary file 1 [file Table1.docx]

**Supplementary Table S1.** Comparison of laboratory examinations of patients with and without cardiotoxicity

|  | **Non-cardiotoxicity group (n = 219)** | **Cardiotoxicity group (n = 81)** | P |
| --- | --- | --- | --- |
| **Renal function** |  |  |  |
| serum creatinine (umol/L) | 55.60 (47.25, 68.45) | 60.00 (50.50, 71.30) | 0.099 |
| blood urea nitrogen (mmol/L) | 4.95 (3.94, 6.30) | 5.18 (3.85, 6.42) | 0.799 |
| **Cardiac function** |  |  |  |
| NT-proBNP ( pg/mL) | 48.00 (25.00, 76.00) | 49.00 (23.00, 67.00) | 0.419 |
| troponin(ng/L) | 4.00 (2.00, 6.00) | 4.00 (2.00, 7.00) | 0.833 |
| Myohemoglobin (ng/mL) | 25.00 (17.00, 32.00) | 24.00 (18.00, 33.00) | 0.748 |
| lactic dehydrogenase (U/L) | 190.00 (160.50, 226.00) | 187.00 (160.00, 246.00) | 0.769 |
| alpha-hydroxybutyric dehydrogenase (U/L) | 127.50 (107.00, 159.00) | 127.00 (110.00, 162.00) | 0.545 |
| creatine kinase (U/L) | 50.50 (35.00, 79.00) | 57.00 (38.00, 82.00) | 0.477 |
| Creatine Kinase Isoenzyme (U/L) | 12.00 (10.00, 16.00) | 12.00 (10.00, 16.00) | 0.627 |
| LVEF | 60.00% (59.00%, 61.00%) | 60.00% (59.00%, 61.00%) | 0.589 |
| **Blood routine** |  |  |  |
| Hemoglobin (g/L) | 117.00 (100.00, 130.00) | 113.00 (100.00, 124.00) | 0.163 |
| blood platelet (^*^10^9^/L) | 164.00 (122.00, 207.00) | 164.00 (128.00, 212.00) | 0.704 |
| white blood cell (^*^10^9^/L) | 4.63 (3.61, 6.14) | 4.90 (3.26, 6.84) | 0.986 |
| **Liver function** |  |  |  |
| glutamic-pyruvic transaminase (umol/L) | 17.00 (11.00, 28.00) | 17.00 (12.00, 27.00) | 0.844 |
| glutamic oxalacetic transaminase (umol/L) | 25.00 (19.00, 34.00) | 24.00 (19.00, 32.00) | 0.552 |
| total bilirubin (umol/L) | 12.60 (9.85, 16.35) | 11.90 (9.20, 15.10) | 0.234 |
| direct bilirubin (umol/L) | 2.50 (1.90, 3.70) | 2.40 (1.80, 3.40) | 0.519 |
| indirect bilirubin (umol/L) | 10.00 (7.60, 13.40) | 9.10 (7.40, 11.90) | 0.180 |
| Albumin (g/L) | 38.00 (34.65, 40.95) | 37.40 (34.40, 40.60) | 0.312 |
| **Electrolyte** |  |  |  |
| Potassium (mmol/L) | 3.90 (3.58, 4.19) | 3.83 (3.67, 4.08) | 0.691 |
| Sodium (mmol/L) | 140.10 (138.35, 141.80) | 139.70 (138.50, 141.10) | 0.392 |
| Calcium (mmol/L) | 2.25 (2.17, 2.33) | 2.22 (2.13, 2.34) | 0.462 |
| Magnesium (mmol/L) | 0.86 (0.81, 0.92) | 0.87 (0.81, 0.92) | 0.761 |

**Supplementary Table S2.** Result of collinearity

| **Factor** | **Tolerance** | **VIF** |
| --- | --- | --- |
| Hypertension | 0.898 | 1.114 |
| Hyperlipidemia | 0.936 | 1.069 |
| Diabetes | 0.912 | 1.097 |
| Age | 0.966 | 1.036 |
| Fluorouracil Drugs | 0.945 | 1.058 |
| Combined Radiotherapy | 0.985 | 1.015 |
